# Clinical benefit of a precision medicine based approach for guiding treatment of refractory cancers

**DOI:** 10.18632/oncotarget.10606

**Published:** 2016-07-15

**Authors:** Milan Radovich, Patrick J. Kiel, Stacy M. Nance, Erin E. Niland, Megan E. Parsley, Meagan E. Ferguson, Guanglong Jiang, Natraj R. Ammakkanavar, Lawrence H. Einhorn, Liang Cheng, Mehdi Nassiri, Darrell D. Davidson, Daniel A. Rushing, Patrick J. Loehrer, Roberto Pili, Nasser Hanna, J. Thomas Callaghan, Todd C. Skaar, Paul R. Helft, Safi Shahda, Bert H. O’Neil, Bryan P. Schneider

**Affiliations:** ^1^ Indiana University Health Precision Genomics Program, Indianapolis, IN, USA; ^2^ Indiana University Melvin and Bren Simon Cancer Center, Indianapolis, IN, USA

**Keywords:** precision medicine, personalized medicine, genomics, next-generation sequencing

## Abstract

**Patients and Methods:**

Patients with metastatic solid tumors who had progressed on at least one line of standard of care therapy were referred to the Indiana University Health Precision Genomics Program. Tumor samples were submitted for DNA & RNA next-generation sequencing, fluorescence in situ hybridization, and immunohistochemistry for actionable targets. A multi-disciplinary tumor board reviewed all results. For each patient, the ratio of progression-free survival (PFS) of the genomically guided line of therapy divided by the PFS of their prior line was calculated. Patients whose PFS ratio was ≥ 1.3 were deemed to have a meaningful improvement in PFS.

**Results:**

From April 2014–October 2015, 168 patients were evaluated and 101 patients achieved adequate clinical follow-up for analysis. 19 of 44 (43.2%) patients treated with genomically guided therapy attained a PFS ratio ≥ 1.3 *vs*. 3 of 57 (5.3%) treated with non-genomically guided therapy (*p* < 0.0001). Similarly, overall PFS ratios (irrespective of cutoff) were higher for patients with genomically guided therapy *vs* non-genomically guided therapy (*p* = 0.05). Further, patients treated with genomically guided therapy had a superior median PFS compared to those treated with non-genomically guided therapy (86 days *vs*. 49 days, *p* = 0.005, H.R. = 0.55, 95% C.I.:0.37-0.84).

**Conclusion:**

Patients with refractory metastatic cancer who receive genomically guided therapy have improved PFS ratios and longer median PFS compared to patients who do not receive genomically guided therapy.

## INTRODUCTION

Precision medicine approaches that seek to match genomic aberrations to potential treatment avenues are rapidly reshaping treatment for cancer patients [1-3]. Advances in genomic technology have enabled clinical application of these approaches, where large scale DNA & RNA profiling of cancer genes from small amounts of archival tissue is now possible [4-6]. In patients with advanced cancer where treatment options are limited, genomic sequencing is frequently being used to identify targets which are potentially clinically actionable with FDA approved drugs or clinical trials. In many cases these targets would have been overlooked under standard clinical practice due to the rarity or novelty of the target, or its presence in a cancer lineage not normally associated with the target (e.g. *HER-2* amplification in colon cancer). These observations and the evolution of targeted therapeutics have spurred a paradigm-shift that recognizes molecular drivers as increasingly powerful additions to the traditionally dominated organ and histology-centric therapeutic standard.

While several publications to date have determined that many cancers have targetable pathways that may benefit from a precision medicine approach [7], formal assessment of clinical benefit of these approaches has been lacking. Published case reports demonstrating exceptional responses to targeted therapy explained by key genomic mutations have brought promise to the field [8-10], but analyses of larger cohorts and randomized controlled clinical trials are still needed to help solidify the utility of cancer genomics in clinical practice. Survival analyses of cohorts with mixed cancer lineages (and mixed genomic aberrations) have been fraught with bias secondary to the widely varying nature of expected survival outcomes for different cancer types. In 2010, Von Hoff et al. [11], reported early experience with identifying molecular targets in patients’ tumors to direct treatment and also performed a formal analysis of clinical benefit by calculating the ratio of the PFS of the patients’ genomically-guided therapy divided by the PFS of their prior line of therapy. This approach took into account both the natural history of the patient's individual cancer, as well as the fact that subsequent lines of therapy nearly always lead to declining PFS times. In their analysis, each patient was able to serve as his or her own control. Therefore, a PFS ratio in excess of 1.0 (or more conservatively, in excess of 1.3 to minimize false-positive fluctuations) provided a reference point of activity relative to the last line of therapy.

In April 2014, the Indiana University Health Precision Genomics Program was initiated to offer next-generation sequencing for patients with metastatic refractory or rare solid tumors in order to provide genomically guided treatment recommendations. Decisions regarding therapy were derived from a multi-disciplinary advisory board with consideration given to strength of genomic association, co-morbidities, prior drug exposure, and inherited genetic variants that would predict increased risk from a drug. Patient demographics, tumor characteristics, information on duration of prior therapies, genomic information, and clinical follow-up were prospectively collected for all patients since the inception of the program. Herein, we report our first formal efficacy analysis to determine the clinical benefit of a precision medicine approach in patients treated with genomically-guided therapy *vs*. non-genomically guided therapy.

## RESULTS

### Patient characteristics

**Figure 1:**
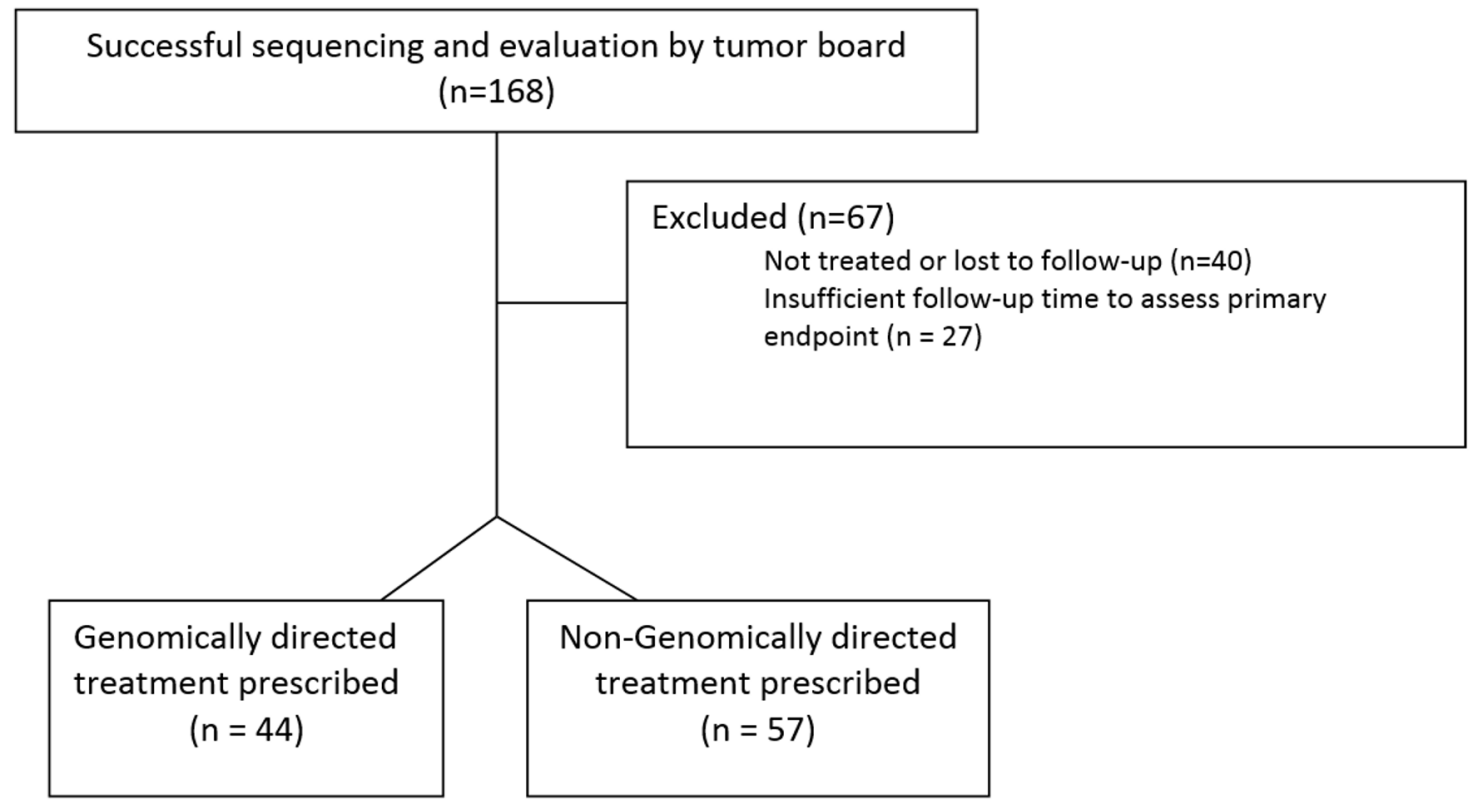
CONSORT diagram of evaluable population

Figure 1 details the Consolidated Standards of Reporting Trials (CONSORT) diagram demonstrating the disposition of the 168 patients who were referred to the Indiana University Simon Cancer Center (IUSCC) Precision Genomics Clinic. Of these, 67 were excluded from the final analysis: the commonest reasons being not receiving any therapy post genomic profiling or lost to follow-up (*n* = 40). Table 1 lists the characteristics of the 101 patients who were referred to the Precision Genomics clinic and are part of the analyzed cohort. Patient demographics were well balanced between groups. The majority of patients had a diagnosis of soft tissue sarcoma, breast cancer, pancreatic cancer or colorectal cancer. The median number of previous therapeutic regimens was 4 for both groups, and 96% of both groups had an ECOG Performance Status (PS) of 0 or 1.

**Table 1:** Patient demographics and clinical characteristics

Characteristics	Genomic directed group (*n* = 44), no. (%)	Non-genomic directed group (*n* = 57), no. (%)	*p*-value
Male Sex	47.7%	45.6%	0.83
Female Sex	52.3%	54.4%	
Mean (+/− SD) Age, years	55.5 (12.5)	58.4 (11)	0.22
ECOG PS			
0	12 (27.3)	16 (28.1)	0.93
1	31 (70.5)	39 (68.4)	
2	1 (2.3)	2 (3.5)	
Mean (+/− SD) time until progression on previous regimen, days	122 (103)	130 (97)	0.75
No. of Prior Regimens			
1 to 2	15	25	0.32
3 to 4	21	24	
5 or greater	8	8	
Median (range) number of regimens	4 (2-6)	4 (2-6)	
Tumor type			
Soft tissue sarcoma	10 (22.7)	11 (19.3)	0.67
Breast	8 (18.2)	8 (14.0)	0.57
Pancreas	1 (2.3)	7 (12.3)	0.13
Colorectal	7 (15.9)	5 (8.8)	0.36
Bladder/Urothelial	2 (4.5)	4 (7.0)	
Prostate	0	1 (1.8)	
Renal cell	1 (2.3)	0	
Cervical	1 (2.3)	0	
Cholangiocarcinoma	1 (2.3)	1 (1.8)	
Esophageal	2 (4.5)	0	
Head and Neck	1 (2.3)	1 (1.8)	
Hepatocellular	0	1(1.8)	
Melanoma	0	3 (5.3)	
Neuroendocrine	1 (2.3)	2 (3.5)	
Non-small cell lung cancer, adeno	0	4 (7)	
Non-small cell lung cancer, squamous cell	0	2 (3.5)	
Small cell lung cancer	1 (2.3)	1 (1.8)	
Squamous cell carcinoma, NOS	0	1 (1.8)	
Unknown Primary	1 (2.3)	0	
Thyroid, anaplastic	1 (2.3)	0	
Adrenal Cortical Carcinoma	0	1 (1.8)	
Small Bowel	1 (2.3)	0	
Ampulla of Vater	0	1 (1.8)	
Chondrosarcoma	1 (2.3)	0	
Endometrial	1 (2.3)	0	
Ewing	1 (2.3)	0	
Glioblastoma multiforme	0	1(1.8)	
Ovarian	1 (2.3)	2 (3.5)	
Myoepithelial	1 (2.3)	0	

### Molecular characteristics

**Figure 2:**
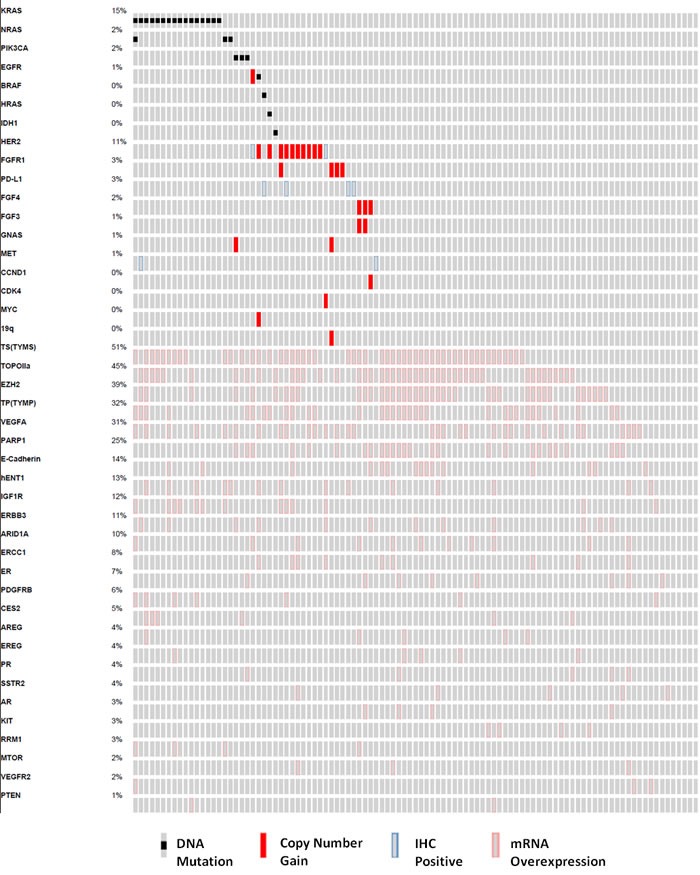
Oncoprint analysis detailing the genomic findings considered by the molecular tumor board for the study population (*n* = 101) Each column represents a single patient, and each row a gene that harbors a mutation, copy number gain, IHC (immunohistochemistry) positivity, or mRNA overexpression.

DNA mutation, copy number variation, Immunohistochemistry (IHC), and mRNA overexpression findings that were considered by the Precision Genomics Tumor Board in determining potential therapies are depicted in Figure 2. The most notable observations that led to clinical interventions included: DNA mutations in *IDH1* and *EGFR*; copy number variation in fibroblast growth factor and Human Epidermal Growth Factor Receptor-2 (HER2); IHC positivity for PD-L1 and MET; and mRNA overexpression for amphiregulin (*AREG*), epiregulin (*EREG*), E-Cadherin (*CDH1*), hENT1, Topoisomerase IIa (TOPOIIa), and Vascular Endothelial Growth Factor-A (VEGFA).

### Progression free survival (PFS) analyses

**Figure 3:**
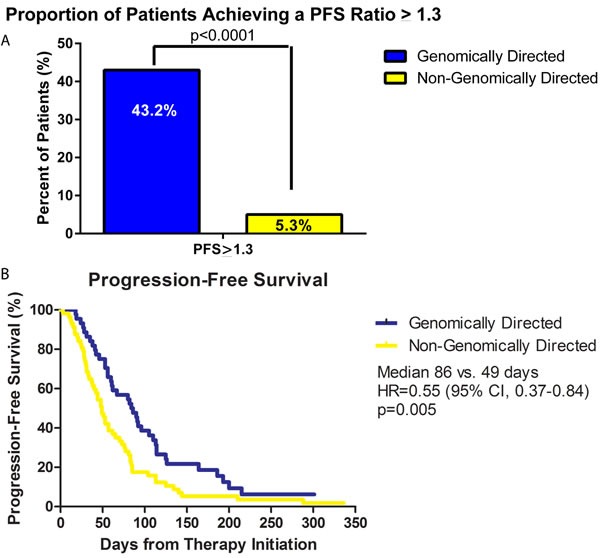
Progression free survival (PFS) analysis of patient population **A.** Bar graph comparing the percentage of patients who achieved a PFS ratio equal to or greater than 1.3. 43.2% (19 of 44) of patients on genomically directed therapy achieved a PFS ratio of 1.3 compared to only 5.3% (3 of 57) who received non-genomically directed therapy (*p* < 0.0001). **B.** Kaplan-Meier plot of the PFS of the genomic-directed group (blue line) *vs*. non-genomic directed group (yellow line). Patients treated with genomic guided therapy had a superior median PFS compared to those treated with non-genomic guided therapy (86 days *vs*. 49 days, *p* = 0.005, H.R. = 0.55, 95% C.I.:0.37-0.84).

43.2% (19 of 44) of patients treated according to genomic recommendations were found to have a PFS ratio of > 1.3 compared to 5.3% (3 of 57) of patients who did not receive treatment guided by genomic recommendations (*p* < 0.0001) (Figure 3A). Patients who did not receive treatment guided by genomic recommendation included the following: a lack of actionable genomic target, inaccessibility to treatment recommendation, or physician choice not to pursue genomic based treatment. A secondary endpoint analysis of PFS ratio >1 (equal to at least the previous regimen) occurred in 50% (22 of 44) and 19.3% (11 of 57) of patients who received genomically directed *vs*. non-genomically directed therapy, respectively (*p* = 0.0011). Similarly, a PFS ratio >1.5 occurred in 29.5% (13 of 44) of patients with genomic directed therapy *versus* 5.3% (3 of 57) without (*p* = 0.0017). When statistically comparing all PFS ratios, patients who received genomically directed therapy had higher PFS ratios compared to non-genomic guided therapy (mean PFS ratio: 1.34 *vs* 0.8, *p* = 0.05). Lastly, Kaplan-Meier PFS analysis demonstrated a median PFS of 86 days in the genomic therapy group *vs*. 49 days in the non-genomic therapy group (*p* = 0.005, H.R. = 0.55, 95% C.I.:0.37-0.84) (Figure 3B).

### Details of responders

**Figure 4:**
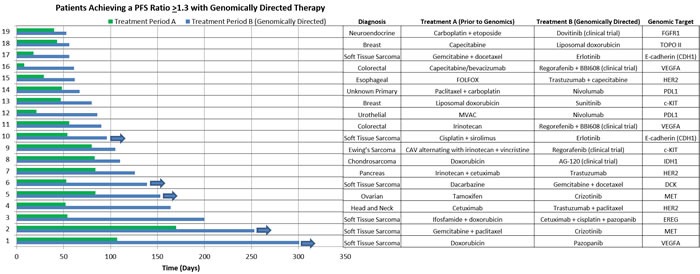
Details of responders Horizontal bar chart showing the PFS duration (days) for those patients in the genomically guided therapy group who achieved a PFS ratio ≥ 1.3 (*n* = 19). In addition, listed is the diagnosis, prior therapy (Treatment A), the genomically directed therapy (Treatment B), and genomic target.

The PFS durations and therapies of those who achieved a PFS ratio ≥ 1.3 (*n* = 19) are detailed in Figure 4. A number of patients with significant benefit had a diagnosis of sarcoma (6 cases with soft tissue sarcoma, 1 case with chondrosarcoma, and 1 case with Ewing's sarcoma). Of interest, five of our patients were continuing a positive response at the time of analysis. Details of the three patients who achieved a PFS ratio of ≥ 1.3 who did not receive genomically-directed therapy are as follows: one patient was diagnosed with NSCLC and received previous therapy with vinorelbine followed by therapy with nivolumab on a clinical trial, the second patient was diagnosed with squamous cancer NOS and received previous therapy with cetuximab followed by therapy with nivolumab on a clinical trial. Of note, both of these patients were seen in our program prior to incorporation of PD-L1 IHC testing as part of our panel. The third patient was diagnosed with metastatic colorectal cancer and was previously treated with regorafenib followed by the non-genomically directed therapy of BBI503 as part of a Phase I clinical trial.

## DISCUSSION

Solid tumors have historically been treated based on the organ site of the cancer's origin. More recently, it has been appreciated that some disease types have important molecular biomarkers that allow for therapies to be tested and used only in a subset of that histological disease subtype [12, 13]. These have been called “actionable targets”. For some disease types, actionable targets are common; for others, they are rare. In the heavily pretreated metastatic setting, conventional cytotoxic therapies have limited success [14, 15]. Many patients in this setting are guided to early phase trials, where novel compounds are being tested with the hope that a unique mechanism of action might provide benefit. The reality of non-molecularly guided early phase trials is that the average response rate and progression free survival is limited [16]. Targeted therapy, conversely, has yielded some examples of substantial benefit when a driver target is appropriately disrupted. Further, these therapeutic targets have occasionally been found to be successful in some tumor types (HER2+ for both breast and gastric cancer) [12, 13, 17] but not others (HER2 in lung cancer) [18]. The incomplete dominance of a therapeutic target implies a complex interaction between the target itself and the disease type, perhaps due to convergent phenotypes or because of the degree of driver dependence and is a major hurdle that must be considered [19].

Affordable and fast high-throughput genomic testing has made it possible to evaluate hundreds or even thousands of driver aberrancies simultaneously. This approach allows for the exploitation of a cadre of drugs including both FDA approved drugs and targeted agents in development in clinical trials. Several trials have been conducted and multiple others are underway to more formally explore the relative success for matching drugs with high throughput genomic biomarkers [20-22]. Although these trials will represent critical steps toward answering the question of the relative efficacy of this approach, to date, these trials have been difficult to execute for a variety of reasons. First, the panel of potential markers and drugs is ever changing and expanding. By the time a trial is complete, the biomarkers and targeted drugs are often outdated. In fact, none to date will have comprehensively included all FDA-approved drugs and all drugs in development. Second, many of these trials have conventional exclusion criteria that don’t mirror the real world scenario where mild organ dysfunction and declining performance status are commonplace in advanced cancer patients. There has also been the creation of clinical programs, similar to that described here, with the goal of maximizing informed decisions. These clinical programs often represent a more generalizable population and may have access to broader selection of therapeutic options.

Despite the advantages highlighted in this type of clinical program, there are several limitations to our study. First, as this was not a randomized-controlled trial, therapeutic selections were recommended (not mandated) and rigorous follow-up with predefined time-points of evaluation were not uniform. Second, targeted genomic sequencing might serve as a prognostic marker rather than a predictive marker. It may be that patients who don’t have actionable targets have more biologically aggressive or resistant disease. Alternatively, those who didn’t receive directed therapy may have also been those patients who were unable to travel to a clinical trial or tolerate therapy because of declining performance status. Lastly, a proportion of those patients not included in the analysis were due to the inability to completely quantify the duration of prior response. This is a unique challenge that traditional clinical trials don’t encounter as the primary measures of response happens during (ORR, PFS) or after enrollment (OS) as opposed to the need to accurately quantify prior duration of response retrospectively. Strengths of this study include the use of a multidisciplinary tumor board to optimize consistency of drug-target selection as well as having a body of experts to routinely review the biomarker literature and evolve as biomarker data and drugs/trials become available in real time. Another strength is the meticulously curated data from the previous treatment experience for each patient and the ability to prospectively follow outcomes.

In our cohort of patients, we found a significant improvement in PFS ratio regardless of cut-point and an improvement in median PFS for the genomically directed group. These findings are congruent with those reported to date and support the evolving body of literature that demonstrates superior outcomes for patients who receive genomically guided therapy [23-25]. Moving forward, programs like ASCO's Targeted Agent and Profiling Utilization Registry (TAPUR) study (http://www.asco.org/practice-research/targeted-agent-and-profiling-utilization-registry-study) will likely facilitate the speed with which the cancer genomics community is able to determine those target/drug combinations with the most utility and simultaneously those that will be futile. Further, the complex interaction between successful target/drug combination and site of disease can be further elucidated with combined data from multiple collaborating groups. Given the emerging body of consistent data demonstrating similar relative benefits to those patients who have access to targeted sequencing, this should be discussed with appropriate patients as a therapeutic avenue.

## MATERIALS AND METHODS

### Study objectives

The primary objective for the study was to compare the frequency of a PFS ratio ≥ 1.3 between patients who received the precision genomics recommended treatment and those who did not. Utilizing the most recent therapy selected by molecular profiling (MP) or clinicians choice of a patient's tumor (Period B) with the PFS for the most recent therapy on which the patient had just experienced progression (Period A). If the (PFS of period B/PFS of period A) ratio was ≥ 1.3, then the tumor board MP-selected therapy was defined as beneficial for the patient. Secondary objectives included mean PFS ratio, PFS ratio ≥ 1, PFS ratio ≥ 1.5, and median PFS between groups.

### Study design

The hypothesis for this study was that using a multidisciplinary tumor board to provide treatment recommendations based on each patient's tumor MP data would favorably change the clinical course for an individual patient. This single institution, prospective, cohort study was conducted in patients with refractory metastatic cancer to compare the PFS of patients receiving a genomic-guided therapy to a cohort receiving a non-genomic guided therapy. The PFS ratio was determined by dividing the PFS of the new therapy (either the genomically guided or the non-genomically guided) by the PFS for the patient during their most recent regimen on which the patient had experienced progression. Patients were evaluated by the Indiana University Health Precision Genomics Program on a referral basis. The study was conducted in accordance with the Declaration of Helsinki and was approved by the Indiana University Institutional Review Board.

### Patient eligibility

Eligibility criteria included the following: any histologic type of metastatic cancer; progression by RECIST criteria on at least one prior regimen for advanced disease; ability to undergo a biopsy or surgical procedure to obtain tumor or having recent tissue available; age > 18 years; referred to the IUSCC Precision Genomics clinic between April 2014-October 2015; Eastern Cooperative Oncology Group performance status of 0 to 2; measurable or evaluable disease; resistance to last line of therapy (documented disease progression under last treatment; discontinued last treatment for progression); and received treatment following Precision Genomic assessment. Patients were excluded if the immediate previous therapy was received on a Phase I or II clinical trial as this could positively bias the PFS ratios if the experimental agent was not active. All patients who did not go on to receive additional therapy (i.e. died or were referred for hospice with best supportive care) were removed from the analysis as to not bias against the non-genomically guided arm and to be able to calculate PFS ratios that can be compared to the genomically guided arm. Minimum follow-up was defined as either disease progression, or the minimum amount of time for a patient to achieve a PFS ratio of 1.3.

### Molecular profiling of patient samples

Formalin-Fixed Paraffin Embedded (FFPE) blocks from tumor biopsies or surgical resections were sent to Paradigm Diagnostics (Ann Arbor, MI) for the PCDx assay. PCDx is a comprehensive next-generation sequencing test that has been designed to analyze most known clinically actionable genomic variations in cancer, including: mutations, gene-fusions/rearrangements, copy number variations, mRNA expression as well as protein expression by IHC. Differences in RNA expression were measured by comparison of tumor gene expression compared to a panel of lineage-matched normal tissues. Next-generation sequencing was performed on an Ion Torrent Personal Genome Machine, with an average coverage > 5,000X. All analyses were performed in a CLIA-certified laboratory. Of note, one patient in our study received testing using the FoundationOne test, whose methods are described elsewhere [26].

### Deliberation of genomic results

A multidisciplinary tumor board of medical oncologists, genomic and pharmacogenomic scientists, oncology clinical pharmacists, an oncologist-ethicist, pathologists, and nurses reviewed the results of the genome analyses. The results were considered in the context of the patient's prior treatment history, concomitant medications, comorbidities, and germline variants that might predict increased toxicity to a given therapy. Identified targets were chosen based on: first priority, DNA mutation or copy number variation or DNA fusions; second priority, IHC or FISH; and last priority, mRNA overexpression alone. Board recommendations were provided to the referring clinician and the patient was treated according to the treating physician's standard-of-care usage of that agent or referred to an available clinical trial.

### Statistical considerations and methods

Using estimates from prior published data [11], a sample size of 74 patients was required to test the hypothesis that the PFS ratio > 1.3 in 28% of patients receiving recommended MP treatment, with an α risk of 5% and a power of 80%, compared to the assumption that the rate of obtaining a PFS of >1.3 without MP guided treatment was 5%. Time-to-event data were summarized using the Kaplan-Meier method and compared using the log-rank test; continuous data were compared using student's t-test; categorical data were compared using the chi-squared or Fisher's exact test, as appropriate.

## References

[1] Roychowdhury S, Iyer MK, Robinson DR, Lonigro RJ, Wu YM, Cao X, Kalyana-Sundaram S, Sam L, Balbin OA, Quist MJ, Barrette T, Everett J, Siddiqui J, Kunju LP, Navone N, Araujo JC (2011). Personalized oncology through integrative high-throughput sequencing: a pilot study. Science translational medicine.

[2] Garraway LA, Verweij J, Ballman KV (2013). Precision oncology: an overview. J Clin Oncol.

[3] Collins FS, Varmus H (2015). A new initiative on precision medicine. N Engl J Med.

[4] Van Allen EM, Wagle N, Stojanov P, Perrin DL, Cibulskis K, Marlow S, Jane-Valbuena J, Friedrich DC, Kryukov G, Carter SL, McKenna A, Sivachenko A, Rosenberg M, Kiezun A, Voet D, Lawrence M (2014). Whole-exome sequencing and clinical interpretation of formalin-fixed, paraffin-embedded tumor samples to guide precision cancer medicine. Nat Med.

[5] Singh RR, Patel KP, Routbort MJ, Reddy NG, Barkoh BA, Handal B, Kanagal-Shamanna R, Greaves WO, Medeiros LJ, Aldape KD, Luthra R (2013). Clinical validation of a next-generation sequencing screen for mutational hotspots in 46 cancer-related genes. The Journal of molecular diagnostics.

[6] Hovelson DH, McDaniel AS, Cani AK, Johnson B, Rhodes K, Williams PD, Bandla S, Bien G, Choppa P, Hyland F, Gottimukkala R, Liu G, Manivannan M, Schageman J, Ballesteros-Villagrana E, Grasso CS (2015). Development and validation of a scalable next-generation sequencing system for assessing relevant somatic variants in solid tumors. Neoplasia.

[7] Kandoth C, McLellan MD, Vandin F, Ye K, Niu B, Lu C, Xie M, Zhang Q, McMichael JF, Wyczalkowski MA, Leiserson MD, Miller CA, Welch JS, Walter MJ, Wendl MC, Ley TJ (2013). Mutational landscape and significance across 12 major cancer types. Nature.

[8] Wagle N, Emery C, Berger MF, Davis MJ, Sawyer A, Pochanard P, Kehoe SM, Johannessen CM, Macconaill LE, Hahn WC, Meyerson M, Garraway LA (2011). Dissecting therapeutic resistance to RAF inhibition in melanoma by tumor genomic profiling. J Clin Oncol.

[9] Wagle N, Grabiner BC, Van Allen EM, Amin-Mansour A, Taylor-Weiner A, Rosenberg M, Gray N, Barletta JA, Guo Y, Swanson SJ, Ruan DT, Hanna GJ, Haddad RI, Getz G, Kwiatkowski DJ, Carter SL (2014). Response and acquired resistance to everolimus in anaplastic thyroid cancer. N Engl J Med.

[10] Wagle N, Grabiner BC, Van Allen EM, Hodis E, Jacobus S, Supko JG, Stewart M, Choueiri TK, Gandhi L, Cleary JM, Elfiky AA, Taplin ME, Stack EC, Signoretti S, Loda M, Shapiro GI (2014). Activating mTOR mutations in a patient with an extraordinary response on a phase I trial of everolimus and pazopanib. Cancer Discov.

[11] Von Hoff DD, Stephenson JJ, Rosen P, Loesch DM, Borad MJ, Anthony S, Jameson G, Brown S, Cantafio N, Richards DA, Fitch TR, Wasserman E, Fernandez C, Green S, Sutherland W, Bittner M (2010). Pilot study using molecular profiling of patients’ tumors to find potential targets and select treatments for their refractory cancers. J Clin Oncol.

[12] Piccart-Gebhart MJ, Procter M, Leyland-Jones B, Goldhirsch A, Untch M, Smith I, Gianni L, Baselga J, Bell R, Jackisch C, Cameron D, Dowsett M, Barrios CH, Steger G, Huang CS, Andersson M (2005). Trastuzumab after adjuvant chemotherapy in HER2-positive breast cancer. N Engl J Med.

[13] Romond EH, Perez EA, Bryant J, Suman VJ, Geyer CE, Davidson NE, Tan-Chiu E, Martino S, Paik S, Kaufman PA, Swain SM, Pisansky TM, Fehrenbacher L, Kutteh LA, Vogel VG, Visscher DW (2005). Trastuzumab plus adjuvant chemotherapy for operable HER2-positive breast cancer. N Engl J Med.

[14] Prigerson HG, Bao Y, Shah MA, Paulk ME, LeBlanc TW, Schneider BJ, Garrido MM, Reid MC, Berlin DA, Adelson KB, Neugut AI, Maciejewski PK (2015). Chemotherapy Use, Performance Status, and Quality of Life at the End of Life. JAMA Oncol.

[15] Longley DB, Johnston PG (2005). Molecular mechanisms of drug resistance. J Pathol.

[16] Agrawal M, Emanuel EJ (2003). Ethics of phase 1 oncology studies: reexamining the arguments and data. JAMA.

[17] Bang YJ, Van Cutsem E, Feyereislova A, Chung HC, Shen L, Sawaki A, Lordick F, Ohtsu A, Omuro Y, Satoh T, Aprile G, Kulikov E, Hill J, Lehle M, Ruschoff J, Kang YK (2010). Trastuzumab in combination with chemotherapy *versus* chemotherapy alone for treatment of HER2-positive advanced gastric or gastro-oesophageal junction cancer (ToGA): a phase 3, open-label, randomised controlled trial. Lancet.

[18] Gatzemeier U, Groth G, Butts C, Van Zandwijk N, Shepherd F, Ardizzoni A, Barton C, Ghahramani P, Hirsh V (2004). Randomized phase II trial of gemcitabine-cisplatin with or without trastuzumab in HER2-positive non-small-cell lung cancer. Annals of oncology.

[19] Ashworth A, Lord CJ, Reis-Filho JS (2011). Genetic interactions in cancer progression and treatment. Cell.

[20] Le Tourneau C, Paoletti X, Servant N, Bieche I, Gentien D, Rio Frio T, Vincent-Salomon A, Servois V, Romejon J, Mariani O, Bernard V, Huppe P, Pierron G, Mulot F, Callens C, Wong J (2014). Randomised proof-of-concept phase II trial comparing targeted therapy based on tumour molecular profiling *vs* conventional therapy in patients with refractory cancer: results of the feasibility part of the SHIVA trial. Br J Cancer.

[21] Barker AD, Sigman CC, Kelloff GJ, Hylton NM, Berry DA, Esserman LJ (2009). I-SPY 2: an adaptive breast cancer trial design in the setting of neoadjuvant chemotherapy. Clinical pharmacology and therapeutics.

[22] McNeil C (2015). NCI-MATCH launch highlights new trial design in precision-medicine era. J Natl Cancer Inst.

[23] Parker BA, Schwaederle MC, Schwab RB, Daniels GA, Piccioni DE, Helsten TL, Bazhenova L, Fanta PT, Lippman SM, Kurzrock R (2015). Precision oncology: the UC San Diego Moores Cancer Center PREDICT experience. Journal of Clinical Oncology: 2015 ASCO Annual Meeting Abstract.

[24] Wheler JJ, Yelensky R, Stephen B, Hong DS, Zinner R, Subbiah V, Fu SQ, Karp DD, Falchook GS, Naing A, Tsimberidou AM, Piha-Paul SA, Janku F, Li YL, Lee JJ, Miller VA (2015). Prospective study comparing outcomes in patients with advanced malignancies on molecular alteration-matched *versus* non-matched therapy. Journal of Clinical Oncology: 2015 ASCO Annual Meeting Abstract.

[25] Cobain EF, Robinson DR, Wu YM, Worden FP, Smith DC, Schuetze S, Chugh R, Ramnath N, Schott AF, Hayes DF, Chinnaiyan AM, Talpaz M (2015). Clinical impact of high-throughput sequencing in patients with advanced cancer: Lessons learned from the Michigan Oncology Sequencing Center. Journal of Clinical Oncology: 2015 ASCO Annual Meeting Abstract.

[26] Frampton GM, Fichtenholtz A, Otto GA, Wang K, Downing SR, He J, Schnall-Levin M, White J, Sanford EM, An P, Sun J, Juhn F, Brennan K, Iwanik K, Maillet A, Buell J (2013). Development and validation of a clinical cancer genomic profiling test based on massively parallel DNA sequencing. Nat Biotechnol.

